# Social structure of the harem-forming promiscuous fruit bat, *Cynopterus sphinx*, is the harem truly important?

**DOI:** 10.1098/rsos.172024

**Published:** 2018-02-07

**Authors:** Kritika M. Garg, Balaji Chattopadhyay, Uma Ramakrishnan

**Affiliations:** 1 Ecology and Evolution, National Centre for Biological Sciences, TIFR, Bellary Road, Bangalore 560065, India; 2 Department of Biological Sciences, National University of Singapore, Singapore

**Keywords:** promiscuous, social system and fission fusion

## Abstract

Bats are social animals and display a diverse variety of mating and social systems, with most species exhibiting some form of polygyny. Their social organization is fluid and individuals frequently switch partners and roosting sites. While harem-like social organization is observed in multiple tropical species, its importance is contested in many of them. In this study, we investigated the role of harems in the social organization of the old world fruit bat *Cynopterus sphinx*. Based on regular behavioural observations over a period of 20 months and genetic data from microsatellite markers, we observed that the social organization is flexible, individuals regularly shift between roosts and the social organization resembles a fission–fusion society. Behavioural and genetic analyses suggest that the harems are not strict units of social structure, and the colony does not show signatures of subdivision with harems as behavioural units. We also observed that there was no correlation between individuals with high association index and pairwise relatedness. Our findings indicate that similar to the mating system, the social organization of *C. sphinx* can also be categorized as a fission–fusion society, and hence the term ‘harem’ is a misnomer. We conclude that the social system of *C. sphinx* is flexible, with multi-male multi-female organization, and individuals tend to be loyal to a given area rather than a roost.

## 1. Introduction

Bats are the second most diverse group of mammals [1]. A high proportion of bat species are social and display a diverse array of mating and social behaviours [2,3]. While vagility allows for flexible associations, social group formation in bats is largely driven by factors like mating opportunities, roost availability and distribution of food resource [2]. Understanding bat sociality requires a thorough knowledge of their social interactions, but only a handful of studies have thus far investigated social interactions in bats in the context of associations between individuals [4–16]. Most of these studies observed that social units are flexible and individuals move between groups. Additionally, these studies also revealed that social group sizes in bats can vary from a few individuals to millions of individuals [2,3], and social units can be maintained year-round or for a few days depending on the species [3]. However, most of our knowledge of social associations within bats comes from temperate species that often show a pattern of fission–fusion behaviour [10,17–20]. Comparatively, knowledge of social interactions of polygynous bats of the tropics, especially the Old World tropics, remains sparse and much of our knowledge of these species comes from a rich scientific literature on behavioural and molecular genetic investigation of the social structure, mating system and their consequences in the oriental fruit bat, *Cynopterus sphinx* [21–24]. However, even the literature on the harem-forming *C. sphinx* lacks sufficient information on long-term detailed behavioural observations, a necessary attribute to understand the dynamics of the social system [22].

In this study, we used long-term regular behavioural observations in conjunction with genetic data to quantify the relationships between individuals and characterize the social system of *C. sphinx*. This frugivorous bat roosts in the undersides of palm leaves, crevices of banyan, peepul, palm or coconut trees, flower and fruit cluster of kitul palm, stem of Ashoka trees and even at the underside of the eaves of houses [22,25]. Males often construct tents by chewing the mid-veins of leaves to form a pendulous tent-like structure and females join them [25]. An association of a resident male with one or more females is commonly called a ‘harem’. A colony consists of multiple harems along with solitary males clustered in an area [25]. Harems are considered both a social and mating unit in *C. sphinx* [23–25]. Harems are labile and are maintained throughout the year despite the presence of well-defined mating periods [5]. Available scientific literature suggests that the harems do not play a role in the social organization or the genetic mating system of *C. sphinx* [21,22,26]. While short-term data suggest a resource defence polygyny [24], temporal data spanning more than six seasons strongly reveal a promiscuous mating system in *C. sphinx* [22]. For a harem-based polygynous mating system, the reproductive success of the harem male and the variance in reproductive success depend on harem size [22]. However, long-term genetic data and limited monthly census revealed that harem size could not predict the reproductive success of males and the variance in male reproductive success in *C. sphinx* [22]. Males which were loyal to a colony had a higher reproductive success compared with males which were not a stable part of the colony [26], suggesting that the colony, rather than the harem, is the mating unit in *C. sphinx*. In the present study, we build on these previous efforts by using long-term behavioural observations (weekly census) to more comprehensively assess the social system of *C. sphinx*. Specifically, we investigated (i) roost fidelity of males and females, (ii) the associations between individuals within a colony, (iii) the presence of preferred associations within the colony, and (iv) correlation between genetic relatedness and such associations. We predict that, for a harem-based social system, we should observe high roost fidelity for harem males compared with solitary males and females. Furthermore, we should observe high levels of association between individuals within a harem. By contrast, for a multi-male multi-female society (non-harem like), we should observe low levels of association between individuals within a harem. Throughout the paper, we follow Kappeler & van Schaik's [27] definition for social system and social organization, wherein social organization describes the size, composition and cohesiveness of a society and social structure outlines the social interactions and relationship among individuals.

## 2. Material and methods

### 2.1. Capture and marking

We studied a population of *C. sphinx* at the Indian Institute of Science (IISc), Bangalore, India (12.99° N, 77.59° E) from August 2011 to April 2013. The bats roosting in this campus use both foliage-modified roost (kitual palm, banana leaves and other ornamental palm trees) and man-made structures (window eves). All the bats captured at IISc were considered to be the part of a single colony. We captured individuals at their day roosts using hoop nets and tagged them with colour-coded beaded necklace for visual identification during observations. We used three bead tags for all the individuals. We noted the age, sex, body size (forearm and tibia length), body mass and reproductive status for all the captured individuals. We took 4 mm wing biopsy from both wings away from any major blood vessels for genetic analysis and stored it in 95% ethanol. All sampling protocols were in accordance with the institutional ethics.

### 2.2. Behavioural observations

Regular weekly census of the colony was carried out by K.M.G. from the last week of August 2011. Individuals were identified based on the three bead colour-coded tags using binoculars (Nikon). For every observation, we recorded the identity of individuals at each roost. For all analyses, we only included those adults who were observed at least twice during the study period.

### 2.3. Genetic data

We genotyped all the individuals at eight microsatellite loci following Garg *et al.* [22,26] and for each locus calculated summary statistics like the number of alleles, observed and expected heterozygosities and polymorphic information content in cervus v. 3.0.7 [28,29]. We used coancestry v. 1.0.1.7 software [30] to estimate relatedness between individuals. As relatedness estimates are dependent on the population structure and the quality of genetic data, we performed simulations in coancestry software to determine the best relatedness estimator. We simulated the following relationships: full-sibs, half-sibs, first, second cousins and unrelated. One hundred dyads each of the above relations were simulated allowing for 1% error in genotyping. To determine the best estimate, we calculated correlation between the true value and seven estimates of relatedness implemented in coancestry.

### 2.4. Analyses

#### 2.4.1. Roost fidelity

We calculated roost fidelity as the maximum proportion of time an individual was identified at a particular roost. During the study period, most individuals occupied multiple roosts. Thus, we only considered the maximum time spent by a bat at any roost as its roost-fidelity index. We also calculated the number of roosts used by each individual and tested whether both roost fidelity and number of roosts used were significantly different between males and females. We used the non-parametric Wilcoxon rank sum test with continuity correction, as the data were not normally distributed. All tests were performed in R v. 2.15 [31]. To compare our results with published results on short-term roost usage and fidelity, we divided our data into smaller time intervals (two months) and estimated an average number of roosts used and average roost fidelity.

#### 2.4.2. Associations

We calculated the half-weight association index (HWI) to determine if individuals associated more than random in socprog v. 2.4 [32]. The HWI is an estimate of the proportion of days pairs roosted together relative to the total number of days each individual in a pair was observed, whether together or separate. The HWI is considered a better estimate for calculating association strength when all individuals with a group cannot be identified [32,33]. To determine whether the observed HWI differed from random expectations, we compared the coefficients of variation (CV) for observed and random association matrices of all possible pairwise associations [34,35]. We generated random matrices by permuting the observed matrices, where pairwise associations were altered but the total number of individuals and the number of groups from the original matrix were conserved [34,36,37]. Associations were considered non-random and significant if the CV of the observed matrix was greater than the random CV in more than 95% of the permutations (*p* > 0.95). In addition, we used socprog to obtain a measure of correlation between the estimated association indices and the true pattern. We also obtained an estimate of social differentiation (*S*) and the average number of associations per individual (*H*) to ensure that the data were sufficient to reject the null hypothesis. If *S*^2^ × *H* > 5, the data have enough power to differentiate between random and non-random associations [38]. Furthermore, we performed Mantel tests in socprog (1000 permutations) to determine whether the sex of the individuals affects the strength of associations.

In addition, we also determined if there were any significant preferred/non-preferred dyadic pairs. We used ‘permute associations within samples' to determine the significant pairs in socprog (*p*-value for preferred associations greater than 0.95 and *p*-value for non-preferred associations less than 0.05).

#### 2.4.3. Cluster diagram

We generated cluster diagrams for the calculated HWI to determine if there was any significant sub-structuring within the data. Analysis was performed in socprog. We used average linkage clustering analyses to create dendograms that linked individuals based on HWI. Individuals with higher HWI cluster together. The clusters are considered distinct groups if the cluster coefficient was equal to or greater than twice the randomly permuted mean. To determine if the cluster diagram was a good representation of the data, we estimated the cophenetic correlation coefficient, which is the correlation between pairwise HWI and the dendogram linkages between pairs [32].

#### 2.4.4. Grouping

We also tested if there were any significant groups within the dataset using the modularity test in socprog. We carried out 10 000 permutations, to determine the modularity. Modularity value greater than 0.3 suggests significant subdivision within the dataset [39].

#### 2.4.5. Network analysis

Network analysis provides an analytical framework for linking individuals based on behaviour. Within a social network, nodes represent individuals, and associations among individuals are represented by valued edges or connections [40]. We used weighted networks, which assign values to edges according to estimated proportion of time individuals spent together based on HWI values. We used netdraw v. 2.158 [41] to graphically illustrate a spring-embedded network, which arranges individuals based on association index. Individual pairs with a higher HWI are closely linked compared with pairs with a lower HWI. We calculated the clustering coefficient (measure of how well the associates of an individual are themselves associated) and affinity (measure of the strength of its associates, weighted by the association index between them) of the network in socprog [32]. The network metrics were averaged across individuals, as they are sensitive to missing data [40,42]. We performed 10 000 randomizations to test if the observed network was non-random in nature.

### 2.5. Nature of associations: effect of relatedness

We preformed mantel tests in socprog to test if related individuals associated more often than others. We performed 10 000 randomizations. We also tested if there was any significant correlation between relatedness and association only for pairs with a significant HWI in R. We also performed bootstrap tests to determine if the average relatedness of pairs with a significant HWI was greater than any random pair. We performed 10 000 bootstraps for the above test in R.

## 3. Results

### 3.1. Capture details

A total of 134 bats were captured from August 2011 to April 2013 including recaptures (number of recaptures = 21, number of unique individuals = 113) (electronic supplementary material, table S1). All individuals were genotyped at eight microsatellite loci. A total of 52 observations were carried out during the study period, out of which 47 observations were considered for all analysis. Five observations were not considered for any analysis, as the bats were disturbed and moved between roosts within the colony during the observation period. The average number of observations per bat was 5.73 (±7.46, standard deviation; range 1–33). We had two or more observations for 67 adult individuals and 50 out of those were observed with other identified individuals. For all analyses based on socprog, we included 50 adults observed with other identified individuals.

As mentioned previously, the bats at IISc used a variety of natural and artificial roosts. Out of 147 eves at IISc, bats occupied 54 window eves at least once during the study period. Twenty-two eves were occupied on a regular basis during the study duration. Four kitul palms (fruit bodies) and three ornamental palms were modified as roosts. These roosts were constructed before the study began. We did not observe any new roost constructions by males. Rarely more than one adult male occupied the same roost. The average harem size was 3.3 (SD: 3.52; range: 1–23).

### 3.2. Genetic diversity and relatedness

We genotyped all individuals (*n* = 113; *n* males = 43; *n* females = 69; sex not recorded for one individual) for eight microsatellite loci and there were no missing data. For adults (*n* = 81), the average number of alleles was 8.3, average observed heterozygosity was 0.75 and polymorphic information content for the loci was 0.68 (electronic supplementary material, table S2). Locus CSP2 was not in Hardy–Weinberg equilibrium due to homozygote deficiency (electronic supplementary material, table S2). Based on the simulations, both dyadic ML [43] and Trio ML [30] relatedness estimated had the highest correlation with the true value (electronic supplementary material, table S3). Hence, for all the analyses, we used dyadic ML estimate. As Queller–Goodnight [44] estimate is most widely used, we also repeated all the analysis with Queller–Goodnight estimate of relatedness. We estimated relatedness for adults who were observed at least twice during the study period (*n* = 67). The observed average relatedness for both males (Queller–Goodnight estimate = −0.021; dyadic ML estimate = 0.078) and females (Queller–Goodnight estimate = −0.012; dyadic ML estimate = 0.077) was slightly lower that that reported for other colonies of *C. sphinx* [26].

### 3.3. Roost fidelity

**Figure 1.**
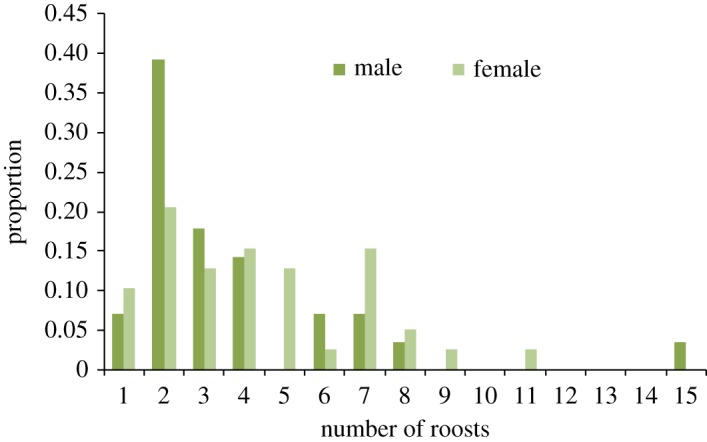
Distribution of the number of roosts used by males and females.

**Figure 2.**
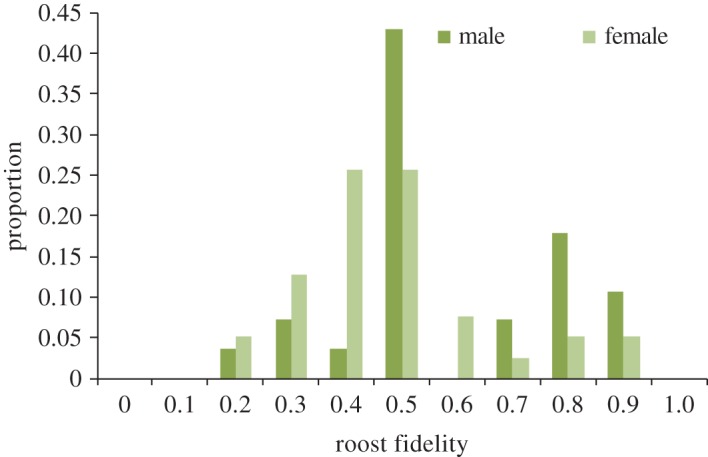
Distribution of roost fidelity for males and females.

We estimated roost fidelity for 67 individuals observed at least twice during the study period. Overall number of roosts used and roost fidelity varied between individuals (figures 1 and 2). Most individuals used multiple roosts (91%). For individuals that occupied more than one roost, the number of roosts used varied between two and 15 (figure 1). We did not observe any significant difference between the number of roosts used by males and females (number of males = 28; number of females = 39; average number of roosts/males = 3.7 ± 2.9; average number of roosts/females = 4.3 ± 2.3; *p-*value = 0.18; figure 1). For individuals that occupied more than one roost, roost fidelity ranged from 0.17 to 0.89. A significant difference was observed between male and female roost fidelity (number of males = 26; number of females = 35; average roost fidelity males = 0.56 ± 1.8; average roost fidelity females = 0.45 ± 1.8; *p-*value = 0.02; figure 2). We also compared roost usage and roost fidelity of harem males with females. Again, there was no significant difference between the number of roosts used by harem males and females (number of males = 22; number of females = 39; average number of roosts/males = 4.2 ± 3.1; average number of roosts/females = 4.3 ± 2.3; *p-*value = 0.39), and the roost fidelity of harem males was significantly higher than that of females (number of males = 21; number of females = 35; average roost fidelity males = 0.56 ± 2.0; average roost fidelity females = 0.45 ± 1.8; *p-*value = 0.03).

Based on the short-term data, the average number of roosts used varied from one to three, and there was no significant difference in the number of roosts used by males and females (number of males = 28; number of females = 39; average number of roosts/males = 1.3 ± 0.4; average number of roosts/females = 1.4 ± 0.5; *p-*value = 0.58). Interestingly, for the short-term data, we did not observe any difference in roost fidelity between males and females (number of males = 14; number of females = 22; average roost fidelity males = 0.82 ± 0.09; average roost fidelity females = 0.77 ± 0.2; *p-*value = 0.47).

### 3.4. Associations

The dataset had enough power to discriminate random associations from non-random ones (*S*^2^ × *H* = (2.92)^2^ × 30.6 = 260.9 > 5; where *S* is an estimate of social differentiation and *H*, the average number of associations per individual), and the estimated associations were a good representation of the true pattern (*r* = 0.92). The observed associations were significantly greater than random (CV for observed associations, 0.78 was higher than that estimated for random associations, 0.64; *p-*value = 0.99, two-tailed test). We did not observe any significant correlation between the sex of the individual and strength of associations (matrix correlation = 0.06; *p-*value = 0.96). Very few individuals had an association greater than 0.1, and non-random associations were observed for 61 pairs (electronic supplementary material, figure S1).

### 3.5. Clustering

**Figure 3.**
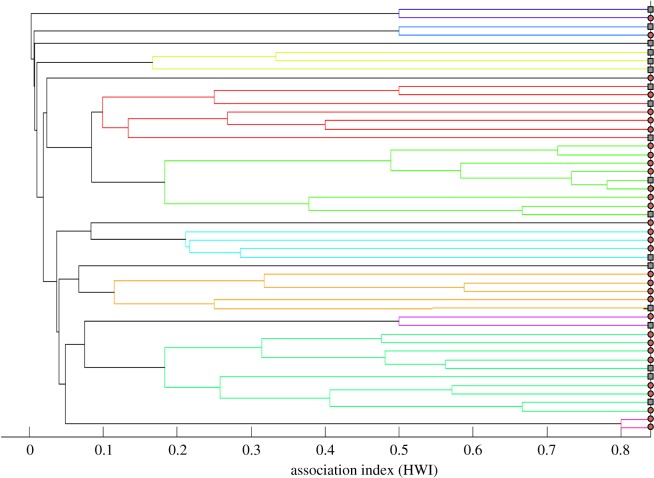
Cluster diagram based on associations for individuals in the colony. Males are indicated in grey squares and brick red circles represent females. Each cluster is indicated by a different colour.

The observed cluster diagram was a good representation of the data as the observed cophenetic coefficient was greater than 0.8 (observed value = 0.83). Although the cluster diagram (figure 3) suggested the presence of groups in the dataset, we did not observe significant clustering as the observed eigenvalue for individual assignment to groups was either negative or near zero, indicating a low confidence in group assignment (electronic supplementary material, table S4). Furthermore, the observed modularity (0.25) was lower than a minimum expected value of 0.3 required for grouping.

### 3.6. Network analyses

**Figure 4.**
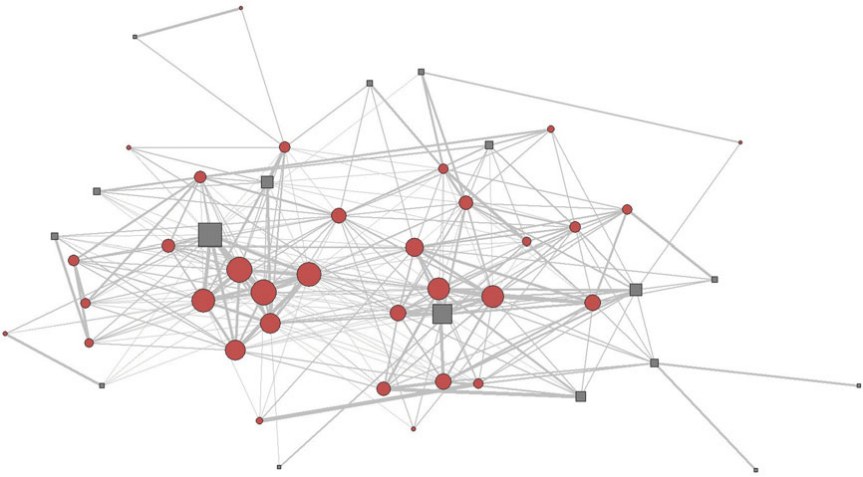
Spring-embedded network diagram for all the individuals with more than two observations. Grey squares represent males and brick red circles represent females. The size of the square/circle denotes centrality for the individual and thickness of the connecting lines indicates the strength of the associations.

Based on the mean clustering coefficient (measure of how well the associates of an individual are themselves associated), the observed network was non-random in nature. The mean clustering coefficient (0.37) for the observed network was significantly greater than expected based on permutations (expected clustering coefficient: −0.13, *p-*value = 0.002) for a random network. Similarly, the observed affinity (0.69) was significantly greater than expected for random associations (expected affinity: −0.003; *p-*value < 0.001). Although the analyses suggest that the network is non-random, qualitatively we did not observe any clusters within the dataset and all individuals were connected to one another (figure 4).

### 3.7. Quality of associations

There was no correlation observed between relatedness and association index between individuals (Dydml: mantel *r* = −0.001, *p-*value = 0.51; Queller–Goodnight: adjusted *R*^2^ = 0.005, *p-*value = 0.44). Furthermore, there was no correlation between relatedness and subset of individuals with significant association index (*n* = 61 pairs, Dydml: adjusted *R*^2^ = −0.01, *p-*value = 0.53; Queller–Goodnight: adjusted *R*^2^ = −0.02, *p-*value = 0.73). Bootstrap tests also revealed no difference in relatedness between all pairs and the subset with a significant association index (Dydml: *p-*value = 0.39; Queller–Goodnight: *p*-value = 0.20)

## 4. Discussion

### 4.1. Harems are not social units

This study supports the idea that *C. sphinx* does not form a stable harem-based social organization (figures 3 and 4; electronic supplementary material, table S4). Our results suggest that both males and females are labile, use multiple roosts (figures 1 and 2) and possibly remain loyal to a given area rather than the roosts, making the social system a flexible multi-male multi-female assemblage. The number of roosts used is comparable between the sexes (figure 1), though males have significantly higher roost fidelity than females (figure 2). Based on a short-term census (38 days), Storz *et al*. [45] too observed similar differences in male and female roost fidelity. However, their study also suggested a significant difference in roost usage pattern [45]. Males rarely shifted roosts compared with females and used up to two roosts [45]. Interestingly we did not observe this trend of roost usage pattern when we subset our dataset into two-month intervals. Although the number of roosts used by males and females varied from one to three in our study colony, there was no significant difference in the number of roosts used by males and females.

Males in *C. sphinx* maintain multiple tents in a given area and bats can switch roosts regularly (A.K. Vinoth Kumar, personal communication). Roost switching in bats is attributed to various factors like access to multiple mates, better transfer of information, familiarity with various roost sites, ease of access to different roost sites, and to reduce predation and parasite infection [46]. However, our study cannot make assumptions in this regard and future studies should address causes and consequences of roost switching in *C. sphinx*. It should also be noted that this study provides an overview of roost usage and fidelity based on weekly census and does not account for daily movement of individuals. Individuals may move between roosts on a daily basis and further studies are required to investigate such movement patterns.

Additionally, our census revealed that adult *C. sphinx* do not preferentially aggregate in harems thereby reducing the importance of tents into roosting sites, and reinforcing the idea the colony is the main social and mating unit [26]. We were able to show by various analyses (cluster, modularity and network analyses) that there is lack of significant grouping in *C. sphinx* (figures 3 and 4; electronic supplementary material, table S4). While our statistical analyses (mean clustering coefficient and affinity) suggested that the network is non-random in nature (figure 4), most individuals were connected with each other, revealing the lack of sub-structuring within our dataset.

Although multiple analyses in this study point towards the instability of harems as roosts, use of multiple roosts and low roost fidelity, these results may be biased due to the type of roosts investigated in the study. *Cynopterus sphinx* uses multiple roost types across its range, from various types of trees to man-made structures [25]. Longevity of natural roosts varies with the type of foliage and man-made structures may provide more permanent roosts to these bats. In IISc, *C. sphinx* uses a mixture of male-modified natural roosts and artificial man-made roost sites (window eves). Roost usage pattern may vary with the type of roosts used as roost fidelity is directly linked to roost longevity [46]. We suggest that comparative studies comprising various roosting habitats may be necessary to understand a general pattern of roost usage and its significance in the social structure and mating system of *C. sphinx*.

### 4.2. Associations are not kin-based

Associations in *C. sphinx* were weak and not correlated with genetic relatedness. Very few individuals had an association index above 0.1 (twice that of random), and out of 1225 possible combinations of associations, only 61 were significant (electronic supplementary material, figure S1), including both preferred and non-preferred associations. Even for the significant association, there was no correlation with genetic relatedness, and the relatedness estimates for pairs with significant HWI were not different from any random pair. Weak social association is common among tent-making bats, such as *Artebius watsoni* [47]. Lack of kin-based association is widespread among bats wherein complex interactions are not kin-based unlike other animal societies [48] (for exceptions, see [11,49]). It seems inclusive that fitness benefits gained by association with kin at roost sites are not an important factor to determine strength of associations in *C. sphinx*.

It is important to note that we estimated association index over the entire duration of the study. Although, in *C. sphinx*, harems are maintained throughout the year, two mating seasons are observed in a year [25]. There might be seasonal variation in levels of association between individuals that our study could not detect due to low number of observations. Future studies should test if seasonal variations in associations are observed in this species.

Furthermore, it should be noted that not all individuals within the colony were tagged and hence we used half-weight index to measure associations, as it is robust to missing data [32,33]. These untagged individuals may be temporary or resident members of the study colony or colonies nearby. Hence, future studies should also compare association and movement of individuals between colonies.

### 4.3. *Cynopterus sphinx* forms multi-male multi-female society

This study highlights the misuse of the term ‘harem’ to define the social structure in *C. sphinx*. In bats, social organization is often characterized as ‘harem’ when single male and multiple females associate together [2,50]. However, the term ‘harem’ has been often criticized recently due to its ambiguous usage [2,50]. For example, the mating system of African banana leaf-roosting bats is promiscuous although their roosting association is still termed as harems [51]. Long-term studies comprising of individual tagging as well as genetic sampling have revealed that similar to *C. sphinx* (this study and other literature), social associations in many social bats are labile and both males and females move across roosts. As a consequence, in many species of bats, temporary harems are now being characterized as multi-male multi-female polygynous groups [2]. For example, both in *Miniopterus minor* and *Tadarida brasiliensis*, the harem-based social system is now characterized as multi-male multi-female units [2]. Stability (long-term maintenance) and reproductive output (harem male sires most of the offspring within the harem) are two important criteria to term the social system as ‘harem based’ [2]. However, neither of these criteria are satisfied in *C. sphinx*. Also, the lack of stable groups within the colony further reduces the support for a ‘harem’-like social structure. Future studies can compare associations between individuals across colonies to confirm the role of colony as a social unit and to understand the movement of individuals across colonies.

In conclusion, observations of this study when viewed in conjunction with our previous enquiry of the social structure and mating system of *C. sphinx* [21,22,26] presents broader implications towards the understanding of individual associations, social structure and mating systems of wild populations. The present study reiterates the importance of a colony-centric view of social structure and mating system in this species. Using the Old World fruit bat *C. sphinx* as a study system, our observations of the social system, in conjunction with our previous conclusions regarding the social and genetic mating system [22,26], highlights the limitations of an anthropocentric ‘harem’-based view of wild populations. It also reiterates the fact that long-term studies can bring into light surprising revelations regarding individual choices, which shapes the dynamics of social structure and mating systems in the wild.

## Supplementary Material

Supporting tables

## Supplementary Material

Figure S1

## Data accessibility

Raw data for this study are available at Dryad Digital Repository (http://dx.doi.org/10.5061/dryad.sp54s) [52].
