# Supplementary material for: Social structure of the harem-forming promiscuous fruit bat, Cynopterus sphinx, is the harem truly important?
Source: R Soc Open Sci. 2018 Feb 7;5(2):172024. doi: 10.1098/rsos.172024 (PMC5830788; doi:10.1098/rsos.172024)
Supplement: Supporting tables [file rsos172024supp1.docx]

TABLES

Table S1: Number of individuals captured in each season.

| Season | Adults (Male/ female) | Pups (attached to the mother/ free) |
| --- | --- | --- |
| Season 1 | 41(15/ 26) | 13 (5/ 7) |
| Season 2 | 32 (12/ 20) | 12 (8/ 4) |
| Season 3 | 29 (12/ 17) | 7 (1/ 6) |

Table S2: Summary statistics for the nine microsatellite markers.

| Locus | Number of alleles | Observed heterozygosity | Expected heterozygosity | Polymorphic information content |
| --- | --- | --- | --- | --- |
| CSP1 | 9 | 0.802 | 0.766 | 0.728 |
| CSP2 | 7 | 0.802 | 0.765 | 0.72 |
| CSP3 | 4 | 0.42 | 0.426 | 0.383 |
| CSP4 | 7 | 0.728 | 0.699 | 0.646 |
| CSP5 | 12 | 0.827 | 0.827 | 0.802 |
| CSP6 | 11 | 0.938 | 0.858 | 0.837 |
| CSP7 | 12 | 0.802 | 0.775 | 0.748 |
| CSP9 | 5 | 0.642 | 0.65 | 0.596 |

Table S3: Correlation between true relatedness values and estimated relatedness.

| Relatedness estimate | Correlation with true estimate |
| --- | --- |
| Trio ML | 0.69 |
| Wang | 0.65 |
| Lynch Li | 0.65 |
| Lynch Rd | 0.61 |
| Ritland | 0.41 |
| Queller Goodnight | 0.64 |
| Dyad ML | 0.69 |

Table S4: Eigen value estimated for each individual to determine significant clusters within the data. Negative value or values near zero indicate lack of confidence in group assignment.

| Individual | Eigen value | Cluster |
| --- | --- | --- |
| 11 | -0.006 | 1 |
| 112 | 0.024 | 1 |
| 13 | -0.007 | 1 |
| 14 | -0.178 | 1 |
| 15 | -0.009 | 1 |
| 44 | -0.258 | 1 |
| 46 | -0.023 | 1 |
| 47 | -0.050 | 1 |
| 50 | -0.004 | 1 |
| 51 | -0.029 | 1 |
| 63 | -0.023 | 1 |
| 65 | -0.011 | 1 |
| 66 | -0.148 | 1 |
| 74 | -0.924 | 1 |
| 136 | 0.002 | 2 |
| 16 | -0.017 | 2 |
| 24 | -0.015 | 2 |
| 25 | -0.053 | 2 |
| 28 | -0.027 | 2 |
| 33 | 0.021 | 2 |
| 34 | -0.007 | 2 |
| 57 | -0.017 | 2 |
| 59 | 0 | 2 |
| 68 | -0.001 | 2 |
| 75 | -0.001 | 2 |
| 78 | -0.005 | 2 |
| 79 | -0.030 | 2 |
| 113 | -0.732 | 3 |
| 119 | -0.176 | 3 |
| 121 | -0.176 | 3 |
| 140 | -0.063 | 3 |
| 64 | -0.546 | 3 |
| 30 | 0 | 4 |
| 31 | 0 | 4 |
| 17 | -0.026 | 5 |
| 18 | -0.011 | 5 |
| 19 | -0.028 | 5 |
| 21 | 0.004 | 5 |
| 22 | 0.014 | 5 |
| 27 | 0.006 | 5 |
| 36 | 0 | 5 |
| 42 | 0.023 | 5 |
| 43 | -0.024 | 5 |
| 69 | 1 | 6 |
| 127 | 1 | 7 |
| 37 | -0.688 | 8 |
| 39 | -0.726 | 8 |
| 110 | 0 | 9 |
| 117 | 0 | 9 |
| 56 | 0 | 9 |
