# Supplementary figures and images for: Social structure of the harem-forming promiscuous fruit bat, Cynopterus sphinx, is the harem truly important?
Source: R Soc Open Sci. 2018 Feb 7;5(2):172024. doi: 10.1098/rsos.172024 (PMC5830788; doi:10.1098/rsos.172024)

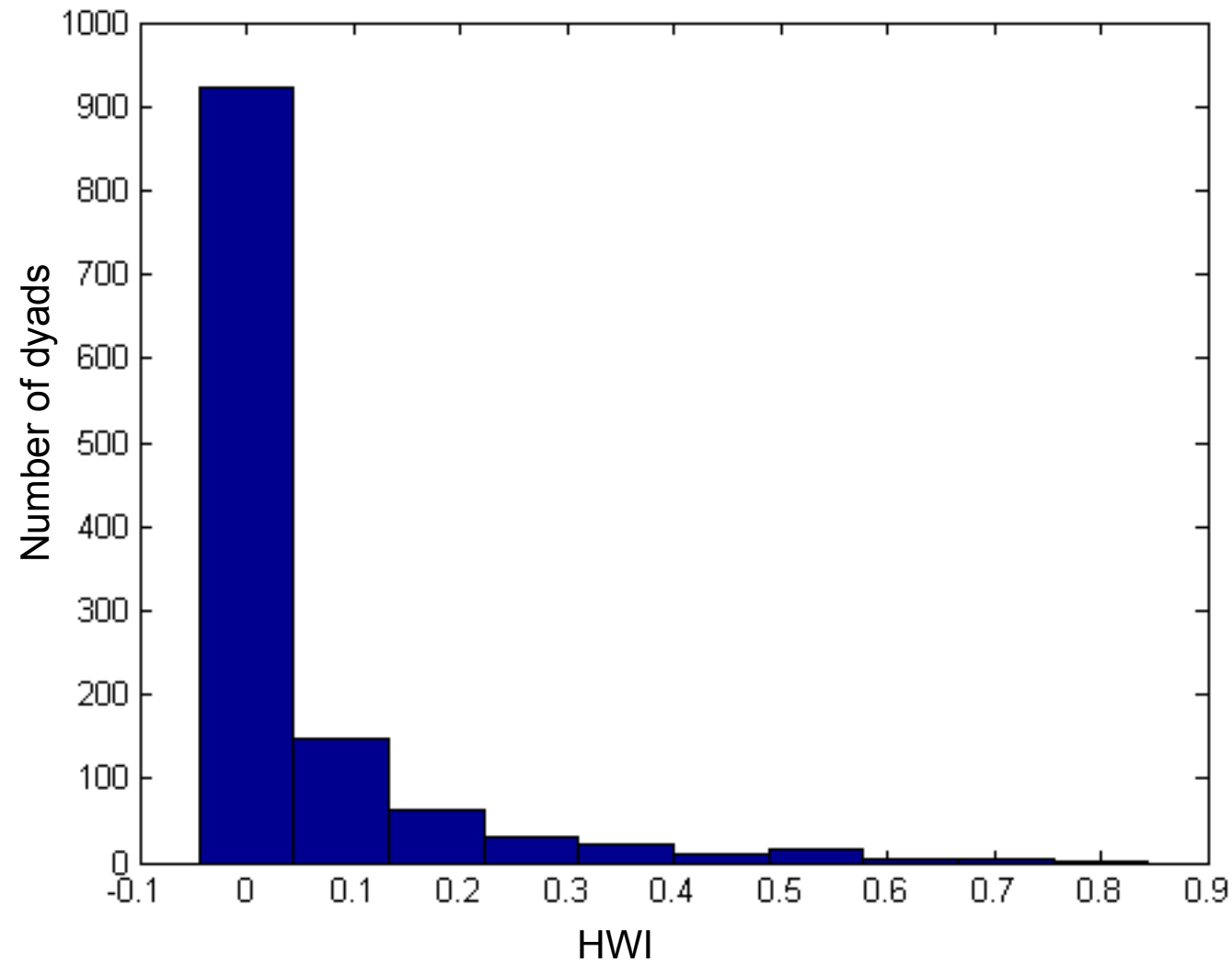

Supplement: Figure S1 [file rsos172024supp2.pdf]
